# Progress in Neurology

**Published:** 1903-01-10

**Authors:** 


### Progress in Neurology.

**The Diagnosis of Functional and Organic Paralysis** is discussed in a most interesting way by Buzzard.[1] By a functional paralysis is meant one which simulates more or less closely those arising from structural alterations of recognised character within the nervous system, without being dependent upon any that the most advanced means of investigation enables us to distinguish. The term should be further limited to cases of paralysis of a kind which it would be possible for at least some healthy persons to produce the appearance of by voluntary simulation. This limitation excludes paralysis confined to the district of a single nerve root or trunk of a cerebro-spinal nerve. Such functional paralysis may have the character of being suddenly or rapidly removed under the influence of persuasion or of some moral or physical shock. Stigmata have no great value, for they may be absent in cases of functional paralysis, and present in undoubted cases of organic diseases. There are many symptoms, the presence of even one of which is conclusive evidence of organic disease; among these are optic neuritis or atrophy, fixed pupil, hemianopsia, absence of knee jerks, reaction of degeneration, "picked out" atrophy of muscles, characteristic bedsores, paralysis of the bladder with ammoniacal urine; paralysis exactly limited to the district of a single nerve or of a plexus, the extensor plantar of Babinski. On the other hand, there are probably no symptoms which can be relied on, unsupported, to establish a diagnosis of functional paralysis. At the onset of a functional hemiplegia there may be a state of unconsciousness present, it is different from apoplexy, however, in that there is no stertor, the face is not flushed, the temperature is unchanged, the pupils respond to light, the deep reflexes are undisturbed, and the patient can be roused. The degree of loss of power varies from time to time, quite unlike the steady passage from flaccidity to contracture of organic hemiplegia. An accompanying complete anæsthesia of the general special senses on one side of the body is almost conclusive evidence of hysteria. If the anæsthesia be partial only, the diagnosis is more difficult. In such cases an important point of difference is that in the organic type hemianopsia may be present, which is never found in hysteria. On the other hand, in hysteria there is amblyopia with concentric contraction of the visual field for light and colour, affecting especially the paralysed side. The gait in the two affections is markedly different; in the organic form the leg is circumducted, in the functional it is dragged. The face is very rarely affected in the functional form; if it is, there is never a lowering of the brow or effacement of the forehead wrinkles, and if the cheek be squeezed between the fingers and passively moved an absence of the muscular relaxation which is observed in organic hemiplegia is noted. A valuable point of distinction is that if the patient be placed on the back and told to try to sit up, it the paralysis is organic the paralysed limb becomes slightly flexed on the trunk whilst the sound limb remains motionless. In functional hemiplegia the limb of the affected side is not lifted. Contracture is the ultimate destiny of all cases of organic hemiplegia; in functional hemiplegia the flaccidity may remain for months or years, or if contracture is present it is found that all the joints can by force be extended at once, whereas in organic hemiplegia no amount of force will straighten the whole limb at once. In exceptional cases of hysteria the tendon jerks may be increased and there may even be clonus, though as a rule this betokens an organic lesion. An absent plantar reflex is almost conclusive evidence of a functional paralysis, whilst an extensor plantar reflex is absolutely certain evidence of organic damage to the pyramidal tracts. In chronic cases of functional paraplegia the limbs may be found generally emaciated, the feet dropped, unable to be dorsiflexed, the joints being stiff apparently from adhesions due to disuse. The resemblance to a transverse lesion of the cord is close, but examination of the knee-jerks, of the electrical reactions of the muscles, and of the condition of the plantar reflex, the presence or absence of an extensor plantar reflex, renders the diagnosis of a crural monoplegia easy; but given a brachial monoplegia in an early stage with absence of anæsthesia there is no certain means of diagnosis, but later on the presence of or absence of contracture effectively distinguishes the causes to which the paralysis may be due. Transitory paralyses may be one of the early features of insular sclerosis, and are often mistaken for hysteria, insomuch as the characteristic features of the disease are not then present. In such cases examination of the plantar reflex often affords most important information—an extensor reflex being certain proof of the presence of organic disease.

**Tabes.**—In a paper on the early manifestations of tabes, Harris [2] calls attention to the fact that the deep reflexes may be exaggerated, and so an error in diagnosis made. This symptom will not decide in favour of general paralysis unless accompanied by more definite signs, such as slurring articulation, tremor, fits, and mental impairment. The worst errors in diagnosis are made in those cases with paroxysmal visceral crises which are often diagnosed as colic, gastritis and gall-stones. When any such attacks are paroxysmal in character, tabes should be thought of. Loss of light reaction of the pupil is undoubtedly the most constant and suggestive sign of tabes, and in conjunction with ulnar or thoracic analgesia, is probably the earliest in the majority of cases ; in some few cases, however, shooting pains and gastric crises many precede them.

[1] Brit. Med. Jour., November 1. [2] Practitioner, October.

(*To be continued.*)

